# Heterogeneous Nuclear Ribonucleoprotein A1 Knockdown Alters Constituents of Nucleocytoplasmic Transport

**DOI:** 10.3390/brainsci14101039

**Published:** 2024-10-19

**Authors:** Todd E. Stang, Hannah E. Salapa, Joseph-Patrick W. E. Clarke, Bogdan F. Popescu, Michael C. Levin

**Affiliations:** 1 Office of the Saskatchewan Multiple Sclerosis Clinical Research Chair, Cameco MS Neuroscience Research Centre, Department of Anatomy, Physiology and Pharmacology, College of Medicine, University of Saskatchewan, Saskatoon, SK S7K 0M7, Canada; tes465@mail.usask.ca; 2 Office of the Saskatchewan Multiple Sclerosis Clinical Research Chair, Cameco MS Neuroscience Research Centre, Department of Medicine, Neurology Division, College of Medicine, University of Saskatchewan, Saskatoon, SK S7K 0M7, Canada; h.salapa@usask.ca (H.E.S.); joseph.patrick.clarke@usask.ca (J.-P.W.E.C.); 3 Cameco MS Neuroscience Research Centre, Department of Anatomy, Physiology and Pharmacology, College of Medicine, University of Saskatchewan, Saskatoon, SK S7K 0M7, Canada; bfp180@mail.usask.ca; 4 Office of the Saskatchewan Multiple Sclerosis Clinical Research Chair, Cameco MS Neuroscience Research Centre, Department of Anatomy, Physiology and Pharmacology, Department of Medicine, Neurology Division, College of Medicine, University of Saskatchewan, Saskatoon, SK S7K 0M7, Canada

**Keywords:** hnRNP A1, nucleocytoplasmic transport, nuclear pore complex, RNA binding protein

## Abstract

Background/Objectives: Changes in nuclear morphology, alterations to the nuclear pore complex (NPC), including loss, aggregation, and dysfunction of nucleoporins (Nups), and nucleocytoplasmic transport (NCT) abnormalities have become hallmarks of neurodegenerative diseases. Previous RNA sequencing data utilizing knockdown of heterogeneous nuclear ribonucleoprotein A1 (hnRNP A1) identified enrichment for pathways and changes in RNAs related to nuclear morphology and showed differential expression of key nuclear targets. This suggests that dysfunction of hnRNP A1, which is observed in neurodegenerative diseases, may contribute to abnormalities in nuclear morphology, NPC, and NCT. Methods: We performed knockdown of hnRNP A1 in Neuro-2A cells, a neuronal cell line, to examine nuclear morphology, NPC, and NCT. Results: First, we examined nuclear morphology using Lamin B, wherein we observed increased nuclear envelope abnormalities in cells with hnRNP A1 knockdown as compared to control. To quantify changes in Lamin B, we designed and validated an automated computer-based model, which quantitatively confirmed our observations. Next, we investigated the impact of hnRNP A1 knockdown on components of the NPC and NCT. In line with the previous literature, we found changes in Nups, including altered distribution and reduced protein expression, as well as disrupted NCT. Finally, we validated our findings in multiple sclerosis (MS) brains, a disease with a significant neurodegenerative component caused by hnRNP A1 dysfunction, where neuronal nuclear envelope alterations were significantly increased as compared to controls. Conclusions: Together, these data implicate hnRNP A1 as an important contributor to nuclear morphology, Nup expression and distribution, and NCT and suggest that hnRNP A1 dysfunction may lead to defects in these processes in neurodegenerative diseases.

## 1. Introduction

RNA binding proteins (RBPs) interact with RNA in a transient or long-term ribonucleoprotein complex [1]. RBPs are involved in many crucial cellular roles, including the processing, modification, splicing, stability, localization, and translation of RNA [2,3]. Dysfunctional RBPs, like heterogeneous nuclear ribonucleoprotein A1 (hnRNP A1), which is highly enriched in neurons, have been associated with neurodegenerative diseases of the central nervous system (CNS), including amyotrophic lateral sclerosis (ALS), frontotemporal dementia (FTD), and multiple sclerosis (MS) [4,5,6,7]. Pathological phenotypes of RBP dysfunction include mislocalization from the homeostatic nuclear location to the cytoplasm and loss of nuclear staining, leading to loss of nuclear function [1,6,8]. Previous research employing RBP cytoplasmic mislocalization mutants and knockdown techniques as models of dysfunction have shown that RBPs, specifically TAR-DNA binding protein-43 (TDP-43), can induce changes in the nucleocytoplasmic transport machinery [9].

The nucleocytoplasmic transport (NCT) machinery encompasses several components, including the nuclear pore complex (NPC), nuclear transport receptors, and the Ran gradient, to regulate efficient transport of molecules across the nuclear membrane. The NPC is a complex containing nucleoporins (Nups), among other proteins, and is formed where the inner and outer nuclear envelopes meet, to allow molecular passage between the nucleus and cytoplasm. There are distinct regions of the NPC, including the asymmetrical nuclear basket that extends into the nucleoplasm and the cytoplasmic ring and filaments which extend into the cytoplasm [10,11]. Symmetrical rings, often referred to as the Y-complex, are located at the periphery on each side of the pore, which allows attachment of the cytoplasmic filaments and nuclear basket [12]. The central structure of the pore includes transmembrane rings embedded in the nuclear envelope and provides structural support and anchoring of the NPC. The Nup93 subcomplex provides further support and scaffolding for the central channel of the pore, which includes proteins with filaments extending into the center of the pore to create a permeability barrier [11,13]. Transport molecules can bind cargo proteins to undergo facilitated diffusion or active transport across the NPC, allowing larger proteins to cross the NPC and accelerate the transport of smaller proteins. 

Initial observations of NCT abnormalities in neurodegenerative diseases were found in ALS, wherein researchers identified irregularities in the NPC and transport [14,15]. These findings have since expanded to include a multitude of morphological and functional defects in the NCT machinery such as changes in the Ran gradient, protein and RNA import defects, changes in distribution of Nups, and nuclear envelope abnormalities [16,17]. Impacts on any one NCT component can initiate changes within another. For example, nuclear envelope lamins are important for NPC distribution and number within the nuclear envelope [18]. Further, alterations in the nuclear lamina and nuclear envelope are closely linked with NCT, wherein the former is causative of defective NCT [19,20]. Conversely, dysfunctional Nups like those that are part of TDP-43 aggregates, impair the structure and function of the NPC, thus impacting NCT [9,17].

The impact of TDP-43 dysfunction in ALS/FTD and tau aggregation on NCT has been explored. However, several other RBPs are dysfunctional in neurodegenerative diseases, including hnRNP A1, which was recently implicated as an important contributor to MS pathogenesis [21]. Several pathways related to cellular transport were found to be altered in systems of hnRNP A1 dysfunction [21,22] and, therefore, we sought to examine the impact of hnRNP A1 dysfunction on the NCT machinery. Herein, we used siRNA-mediated knockdown of hnRNP A1 [22] to model loss of nuclear hnRNP A1 function, a form of dysfunction observed in human tissues [6,8]. First, we found enrichment for NCT machinery-related pathways from an RNA sequencing dataset from differentiated neuronal cells with hnRNP A1 knockdown. Terms including nuclear envelope and lamin were highly enriched, and thus we examined nuclear morphology via Lamin B staining following hnRNP A1 knockdown to identify potential abnormalities. We developed and implemented an automated quantification method based on 3D measurements of Lamin B to determine that hnRNP A1 knockdown significantly increased the number of cells with Lamin B abnormalities. We then examined other components of the NCT machinery. Here, we found significant alterations in Nup98 and POM121, components of the NPC, and RanGAP1, a nuclear transport receptor, following hnRNP A1 knockdown. We further determined that hnRNP A1 knockdown affected active NCT between the nucleus and cytoplasm. We validated our findings in neurons from MS brains, which, like other diseases with NCT machinery alterations [20,23,24], showed abnormalities in Lamin B. These findings establish that loss of hnRNP A1, a form of RBP dysfunction, can disrupt nuclear morphology, components of the NCT machinery, and NCT itself. Therefore, hnRNP A1 dysfunction may contribute to neuronal damage via disruptions to the NCT machinery in neurodegenerative diseases. 

## 2. Materials and Methods

Cell culture and transfection: Neuro-2a cells (Cedarlane Labs, Burlington, VT, Canada, CCL-131), which exhibit a neuronal phenotype upon differentiation, were grown in T-75 flasks at 37 °C in a humidified environment containing 5% CO_2_ and 95% normal atmosphere. Cells were passaged at 80% confluency in complete media, consisting of DMEM with 10% fetal bovine serum (FBS) and 1% penicillin/streptomycin. Neuro-2a cells were differentiated into a neuronal phenotype with differentiation media containing DMEM, 1% penicillin/streptomycin, 2% FBS, and 10 µM retinoic acid. For siRNA experiments, cells were transfected using lipofectamine RNAiMAX (Invitrogen, West Etobicoke, ON, Canada) and 0.0125 µM of siRNA. The efficacy of this siRNA targeting hnRNP A1 in Neuro-2a cells has been previously established [22]. For experiments examining hnRNP A1 knockdown and nucleocytoplasmic transport, co-transfection of siRNA and the plasmid of interest was performed using lipofectamine 2000 (Invitrogen, West Etobicoke, ON, Canada), 400 ng of plasmid, and 0.0125 µM of siRNA. Cells were seeded at 25,000 cells per well on 8-well plates for 24 h prior to transfection; 16 h post transfection, the media was changed to differentiation media as previously published [22], and 72 h after transfection, cells were harvested or fixed for downstream experiments. Nuclear import or export inhibition were achieved using Importazole (Sigma-Aldrich, Oakville, ON, Canada, SML0341) at a concentration of 40 µM and Leptomycin B (Sigma-Aldrich, Oakville, ON, Canada, L2913) at a concentration of 10 ng/mL, respectively, suspended in DMSO and diluted in differentiation media.

siRNA oligonucleotides and plasmids: The siRNA sequence for scrambled/negative control siRNA (siNEG) was 5′-UGGUUUACAUGUCGACAAA-3′, and siRNA targeting A1 (siA1) was 5′-GUAUCCAUUAUCAUGUGUA-3′ [22] both synthesized by Integrated DNA Technologies (IDT). The N-Lentiviral-S-tdTomato-C plasmid was a gift from Jeffrey Rothstein [Addgene plasmid # 112579; RRID: Addgene_112759] [25]. The N-pAAV-hSyn-2xNLS-tdTomato-C plasmid was generated using HiFi DNA assembly cloning (NEBuilder HiFi DNA Assembly Cloning Kit, New England BioLabs, Whitby, ON, Canada). Briefly, the 2xNLS-tdTomato sequence was PCR cloned out of N-pAAV-MBP-2xNLS-tdTomato-C [a gift from Viviana Gradinaru, Addgene plasmid # 104054; RRID: Addgene_104054] [26] using the forward ctgagagcgcagtcgagaagatgggaagcccaaagaag and reverse tatcgataagcttgatatcgtcacaccttccgctttttc PCR primers. The cloned product was inserted into the N-pAAV-hSyn-mScarlet-C plasmid [a gift from Karl Deisseroth, Addgene plasmid # 131001; RRID: Addgene_131001] [27] between the BamHI and EcoRI restriction enzyme sites with two-fragment HiFi DNA assembly cloning. This cloning fully replaced the mScarlet gene cassette with the 2xNLS-tdTomato sequence.

For bacterial growth and plasmid expansion, overnight cultures at 37 °C were inoculated with bacterial stocks suspended in sterile terrific broth (Sigma-Aldrich, Oakville, ON, Canada, 71754) supplemented with the appropriate antibiotic to expand the bacterial numbers. Cells were lysed to isolate plasmids using a PureLink^TM^ HiPure Plasmid Midiprep kit (ThermoFisher K210005, Waltham, MA, USA), following the manufacturer’s protocol. Plasmid concentrations were determined using a Nanodrop ND-1000 spectrophotometer (ThermoFisher).

Cell lysis and protein extraction for Western blotting: Neuro-2a cells were cultured on 6-well dishes coated with poly-d-lysine. Cells were harvested via scraping into Dulbecco’s phosphate-buffered saline (D-PBS) and centrifuged for 5 min at 500× *g*. Pelleted cells were resuspended in CytoBuster (Millipore) containing protease inhibitors (Roche, Mississauga, ON, Canada), rotated, and then centrifuged at 16,000× *g* for 5 min. The resultant supernatant was isolated and stored at −80 °C until Western blot experiments.

Western blotting: Protein lysates were precipitated in acetone at −20 °C for at least 20 min, followed by centrifugation at 18,000× *g* for 20 min. The supernatant was removed, and the pellet was resuspended in 1× sample buffer with β-mercaptoethanol (BME). A total of 40 μg of protein was loaded into each well and separated via SDS-PAGE on a 10% acrylamide gel run at 120 V. Proteins were transferred to PVDF membrane for 30 min at 10 V or overnight as a cold wet transfer at 30 V. Membranes were blocked with 10% normal goat serum for 1 h at room temperature and then incubated with primary antibodies overnight at 4 °C. The following primary antibodies were used: mouse anti-hnRNP A1 (1:1000; Millipore, Oakville, ON, Canada, 05-1521), rat anti-Nup98 (1:1000; Abcam, Cambridge, MA, USA, ab50610), rabbit anti-ß-actin (1:1000; Cell Signaling Technology, Danver, MA, USA, 4967), and mouse anti-ß-actin (1:2000; Cell Signaling Technology, Danver, MA, USA, 3700), rabbit anti-RanGAP1 (1:1000; Abcam, Cambridge, MA, USA, ab92360), and mouse anti-Transportin 1 (1:1000; Novus Bio, Toronto, ON, Canada, NB600-1397). Membranes were washed and incubated with secondary antibody. The following secondary antibodies were used: goat anti-mouse IgG (1:3000; Bio-Rad, Hercules, ME, USA, 1706516), goat anti-rabbit IgG (1:3000; Bio-Rad, Hercules, ME, USA, 1706515) and goat anti-rat IgG (1:9000; Jackson, Bar Harbor, ME, USA, 112-035-003), all conjugated to horseradish peroxidase. Membranes were developed using Clarity Western ECL substrate (Bio-Rad, Hercules, Savannah, GA, USA) for 5 min and visualized using the Bio-Rad ChemiDoc system. Protein levels were quantified using ImageJ by densitometry and normalized to ß-actin.

Immunohistochemistry: Formalin-fixed paraffin-embedded (FFPE) brain tissue (frontal and parietal cortex) from both sexes was obtained from the Netherlands Brain Bank (NBB), Netherlands Institute for Neuroscience, Amsterdam (https://www.brainbank.nl/, accessed December 2020). All material has been collected from donors for, or from whom, a written informed consent for a brain autopsy and the use of the material and clinical information for research purposes has been obtained by the NBB. The 10 µm sections were deparaffinized through 5 min sequential washes of xylene (2×), 50/50 xylene and 100% ethanol, 100% ethanol (2×), and 95% ethanol. Slides were incubated in 0.667% H_2_O_2_ in methanol for 30 min to block endogenous peroxidases followed by 5 min washes in 95% ethanol and 70% ethanol. For antigen retrieval, slides were incubated in Tris-EDTA buffer (pH 9; 10 mM Tris, 1 mM EDTA, 0.05% Tween-20) in a steamer for 45 min. Slides were washed in 0.1M PBS (3 × 5 min) and blocked in 10% FBS in 0.1 M PBS for 15 min. Rabbit anti-Lamin B (1:1000; Abcam ab16048) was incubated overnight at 4 °C, diluted in blocking solution. Slides were washed in 0.1M PBS and incubated with donkey anti-rabbit IgG conjugated to biotin (1:200 Jackson, Bar Harbor, ME, USA, 711-065-152) diluted in 10% FBS and 3% human serum in 0.1 M PBS for 1 h at room temperature. Slides were washed in 0.1 M PBS and incubated with avidin peroxidase (10 µg/mL; Sigma, Oakville, ON, Canada, A3151) diluted in 10% FBS in 0.1 M PBS for 1 h and washed in 0.1 M PBS. Slides were developed in 0.05% 3,3′-diaminobenzidine (DAB) before washing in tap water. Slides were counterstained with hematoxylin for 10 min, rinsed in tap water, destained in 0.5% HCl in 70% ethanol, rinsed with tap water, and placed in Scott solution (0.2% KHCO_3_ and 2% MgSO_4_*7H_2_O in distilled water). Slides were dehydrated in sequential 5-min washes in 70% ethanol, 95% ethanol (2×), 100% ethanol (2×), 50/50 xylene and 100% ethanol, and 100% xylene (2×). Slides were coverslipped using vectamount (Vector Laboratories, Burlington, ON, Canada, H-5700-60) and imaged using an Olympus BX61VS Scanning microscope under a 40X objective, with a numerical aperture of 0.95. Images were processed in Olympus VS-ASW FL 2.7 software, and analysis was performed using QuPath v0.4.3 (see abnormal staining quantification). Representative images were taken using a 100× oil objective, numerical aperture 1.3, on an Olympus BX53 microscope, and processed using Olympus CellSens Standard 1.5 software.

Immunocytochemistry: Neuro-2a cells were cultured in 8-well chamber slides coated with poly-D-lysine (Sigma-Aldrich, Oakville, ON, Canada). Cells were fixed with 3.7% formaldehyde in D-PBS for 15 min at room temperature, washed three times in PBS and permeabilized with 0.1% Triton X-100 in PBS (0.1% PBS-T) for 10 min. Cells were blocked with 5% bovine serum albumin (BSA) in 0.1% PBS-T for 1 h, followed by overnight incubation at 4 °C with primary antibodies diluted in blocking solution. Primary antibodies used included rabbit anti-Lamin B (1:1000; Abcam, Cambridge, MA, USA, ab16048), mouse anti-hnRNP A1 (1:500; Millipore, Oakville, ON, Canada, 05-1521), rabbit anti-hnRNP A1 (1:500; Abcam, Cambridge, MA, USA, ab4791), rabbit anti-POM121 (1:500; Novus, Vancouver, BC, Canada, NBP2-19890), mouse anti-RanGAP1 (1:250; Santa Cruz, Dallas, TX, USA sc-28322), rat anti-Nup98 (1:500; Abcam, Cambridge, MA, USA, ab50610), rat anti-Nup62 (1:500; Millipore, Oakville, ON, Canada, MABE1043), chicken anti-β-III-tubulin (1:500; Aves Lab TUJ, St. Tigard, OR, USA), and rabbit anti-β-tubulin (1:1000; Sigma-Aldrich, Oakville, ON, Canada, T2200). Cells were washed thrice in 0.1% PBS-T and incubated with secondary antibodies for 30 min at room temperature. Secondary antibodies used included donkey anti-mouse Alexa Fluor 488 (1:1000; Jackson Immunoresearch, West Grove, PA, USA, 715-546-151), goat anti-rabbit Alexa Fluor 488 (1:1000; Jackson Immunoresearch, West Grove, PA, USA, 111-545-006), goat anti-mouse DyLight 594 (1:1000; Jackson Immunoresearch, West Grove, PA, USA, 115-515-006), goat anti-rabbit DyLight 594 (1:1000; Jackson Immunoresearch, West Grove, PA, USA, 111-586-006) donkey anti-rat Alexa Fluor 594 (1:1000; Jackson Immunoresearch, West Grove, PA, USA, 712-586-153), and donkey anti-chicken Alexa Fluor 647 (1:1000; Jackson Immunoresearch, West Grove, USA, 703-606-155). Slides stained with the conjugated antibody rabbit anti-hnRNP A1 Alexa Fluor 647 (1:250; Abcam, Cambridge, MA, USA, ab197854) were washed in 0.1% PBS-T three times and incubated overnight at 4 °C overnight diluted in blocking solution. Coverslips were mounted with ProLong Gold antifade reagent with DAPI (Invitrogen, West Etobicoke, ON, Canada) and imaged with a 40× or 63× objective, with a 1.40 numerical aperture, on an Axio Observer 7, inverted compound fluorescent light microscope (Carl Zeiss Canada Ltd., North York, ON, Canada). Images were processed using ZEN 3.1 Blue Edition software (Carl Zeiss Canada Ltd., North York, ON, Canada). Representative images in figures were created from Z-stacks of the marker of interest, followed by deconvolution.

Fluorescence quantification of hnRNP A1 and tdTomato signal: ImageJ was used to measure the fluorescence intensity of stained cells. Regions of interest (ROIs) were generated from corresponding DAPI images to prevent bias and outline the nuclei. The outlined nuclei were overlaid on the fluorescent channel images of interest (hnRNP A1 or tdTomato). The corrected mean nuclear fluorescence was calculated by measuring the mean nuclear fluorescence intensity for hnRNP A1 or tdTomato and subtracting the mean background fluorescence intensity. For 3D- and abnormal-staining quantification, cells were included in analyses if they had less than 50% corrected mean nuclear hnRNP A1 fluorescence compared to the control group. Thus, only cells with greater than 50% hnRNP A1 knockdown were used for analysis. The corrected mean nuclear fluorescence was used to identify individual cells with hnRNP A1 knockdown, as it did not rely on the size of the nucleus, which can be variable in Neuro-2a cells. The corrected total nuclear fluorescence was calculated by taking the integrated density of the ROI and subtracting the area of the nucleus multiplied by the background fluorescence to represent overall hnRNP A1 fluorescence in the siNEG- and siA1-treatment groups. 

In vitro abnormal morphology quantification: Cells treated with siNEG and siA1 were stained for different markers of NCT machinery (Nup62, Nup98, POM121, RanBP2, RanGAP1). Single z-slice images were used to analyze the staining. For each marker, normal and abnormal phenotypes were defined based on previous reports of NCT alterations in neurodegenerative diseases [9,17,25,28,29]. The percent of abnormal phenotypes was calculated by adding the counts of the different abnormal phenotypes and dividing by the total number of cells counted (n = 30–40 cells per replicate per group). The mean percentage of abnormal staining phenotypes between the siNEG and siA1 groups was compared using one-tailed, independent *t*-tests. 

In situ abnormal Lamin B quantification: Whole human brain-tissue slides stained for Lamin B were imaged using an Olympus BX53 microscope equipped with an Olympus DP72 camera. Human brain tissues were analyzed using QuPath v0.4.3 [30]. Tissue section images were loaded into QuPath in the .vsi format. The image type was set to Brightfield H-DAB, and the brightness and contrast were altered appropriately to view the channels separately and to best identify Lamin B phenotypes. A region of interest (ROI) of 2000 pixels by 2000 pixels, created using the Objects → Annotations → Specify Annotation tab, was placed in approximately layer III of the cortical gray matter. Five ROIs were analyzed per tissue section in this area. Within each square, glial cells were identified and removed from analyses to avoid confusion when counting neurons. Neurons were classified as normal or abnormal, based on Lamin B staining, using the counting tool. All samples were renamed using a random letter code to blind cases during analysis. After abnormal phenotypes were counted, samples were unblinded to statistically test the results.

Lamin B three-dimensional quantification: Cells with hnRNP A1 knockdown or siNEG-treated cells were stained for Lamin B. The 3D ImageJ suite plugin for ImageJ was used for 3D quantification [31]. A Z-stack of Lamin B tiff images was combined into a stack in ImageJ, with the distance between images and the scale in the images appropriately calibrated. Images were processed with ImageJ using the optimal auto threshold on the z-plane most in focus and applying the same threshold to all images in the stack. Images were processed again to be compatible with the 3D quantification tool using 3D fast filers in the plugin, using the median method and default settings. The Lamin B staining was outlined, and all individual measurements for a single nucleus were combined and then measured, returning all the values used for 3D quantification. Three replicates were quantified per group. 

Gene ontology (GO) analysis and normalized count values: Differentially expressed genes in Neuro-2a cells with hnRNP A1 knockdown were previously identified through bulk RNA-sequencing analysis and analyzed as previously described [22]. GO pathways were manually examined for terms related to the NCT machinery and representative terms were chosen for graphical representation. Genes from these chosen pathways were identified, and normalized gene counts for each gene of interest from siNEG- and siA1-treated cells were plotted and individually analyzed to identify significant changes in gene expression.

Experimental design and statistical analysis: Graphical representations and data analysis were completed using GraphPad Prism 10 software (GraphPad Software, San Diego, CA, USA). Paired *t*-tests were used to compare hnRNP A1 knockdown, as biological replicates 1, 2 and 3 had variable exposure times (exposure times between treatments in the same replicate were identical), making hnRNP A1 fluorescence dependent on the imaging parameters and, therefore, related (Figure S1). One-tailed independent *t*-tests were used to compare means when the treatment was expected to produce a specific directional effect. Two-tailed independent *t*-tests were used to compare means when the expected treatment effect was unknown. Data were normally distributed, and the variance between samples was assumed to be similar for all samples that had three or less replicates. The percent of abnormal phenotypes in human samples was normally distributed (Shapiro–Wilk W: Control W = 0.9232, *p* = 0.5505; MS W = 0.9811, *p* = 0.9877) and the variance between the two samples was statistically equal (F-test: F_11,4_ = 1.863, *p* = 0.5761). Therefore, an independent *t*-test was used to analyze the samples. G*Power 3.1 was used to complete a power analysis related to Fisher’s exact test to determine the sample size needed to achieve a power of 0.8 using the effect size found in the experiment in Figure S2 [32]. Values in graphs are plotted as the mean ± standard error of the mean (SEM) in all instances, denoted with the statistical test used with and *p* < 0.05 considered statistically significant.

## 3. Results

### 3.1. hnRNP A1 Knockdown Alters the Expression of Genes Related to the NCT Machinery

**Figure 1:**
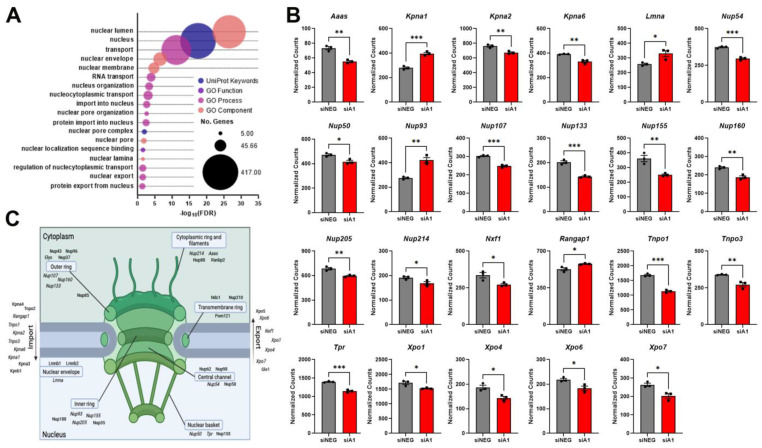
hnRNP A1 knockdown alters mRNA transcripts related to pathways and components of NCT. (**A**) Bubble plot of significantly altered pathways related to the nuclear structure, NPC and NCT identified from GO analyses. The *x*-axis denotes the -log_10_ false discovery rate (FDR) value for each term while the *y*-axis is indicative of each individual GO term. Bubble size corresponds to the number of differentially expressed genes within the GO term and colors correspond to the different GO pathways. (**B**) Individually analyzed genes from the pathways in (**A**) comparing normalized RNAseq count values from siNEG- and siA1-treated cells. (**C**) Summary figure illustrating components of NCT, emphasizing where proteins encoded by genes from (**B**) are located (italicized targets). Created with BioRender.com. Data are graphed as mean ± SEM with n = 3 replicates. As siA1 was expected to produce a specific directional effect, a one-tailed independent *t*-test was used to compare means between siNEG and siA1 in (**B**), * *p* < 0.05 ** *p* < 0.01, *** *p* < 0.001.

Knockdown of hnRNP A1 in differentiated Neuro-2a cells altered the expression of over a thousand genes [22]. Differentially expressed genes were analyzed using gene ontology (GO), which identified numerous pathways related to NCT. Specifically, we found that pathways related to the nuclear envelope, NPC, NCT, nuclear pore, and import/export mechanisms were prevalent (Figure 1A). We examined the genes within these pathways separately from the bulk RNA sequencing dataset (Figure 1B) and determined that some genes, but not all, from components of NCT were changed (Figure 1C). For example, nuclear transport receptors like Tnpo1 and Rangap1 were differentially expressed, as well as Lmna, a component of the nuclear envelope (Figure 1B). We further examined these targets to determine if there were concomitant protein changes following hnRNP A1 knockdown. Here, we found that, in addition to changes in RNA expression, hnRNP A1 knockdown also led to protein-expression level changes in RanGAP1 and Transportin-1 (Figure S1). Interestingly, the major components of the NPC all contained up- or downregulated genes, except for the transmembrane ring (Figure 1C). Therefore, we further investigated components of NCT using siRNA-mediated knockdown targeting hnRNP A1 (siA1).

### 3.2. Knockdown of hnRNP A1 Significantly Alters Nuclear Envelope Morphology

**Figure 2:**
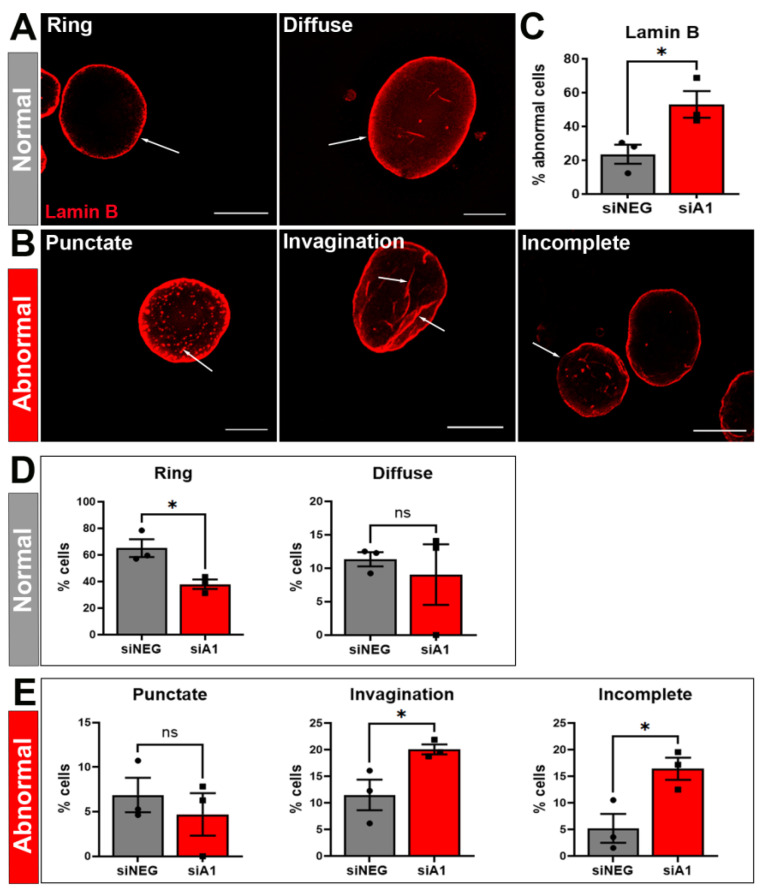
hnRNP A1 knockdown increases the prevalence of abnormal Lamin B phenotypes. Cells were separated into distinct phenotypic categories based on the Lamin B staining pattern. Representative images for each phenotype were generated by deconvolving 40X Z-stack images. (**A**) Ring and diffuse phenotypes were considered normal based on the literature and their prevalence in siNEG-treated cells (**B**) Punctate, invagination, and incomplete phenotypes were considered abnormal, with arrows pointing to the phenotype-defining characteristic. (**C**) Quantification of the number of cells exhibiting normal and abnormal Lamin B phenotypes in siNEG- and siA1-treated cells, with a significant increase in the number of abnormal phenotypes with hnRNP A1 knockdown. (**D**) Assessment of individual normal Lamin B phenotypes demonstrated a significant decrease in ring, but not diffuse, phenotypes between siNEG- and siA1-treated cells. (**E**) Assessment of individual abnormal Lamin B phenotypes identified a significant increase in the invagination and incomplete phenotypes, but not the punctate phenotype, between groups. Data are graphed as mean ± SEM. Scale bars = 10 µm, n = 3 replicates. As siA1 was expected to produce a specific directional effect based on the RNA sequencing data, a one-tailed independent *t*-test was used to compare means between siNEG and siA1 in (**C**–**E**), * *p* < 0.05, ns = not significant.

Our GO analysis identified specific components of NCT that were affected by hnRNP A1 knockdown. The nuclear envelope and nuclear lumen were two enriched GO terms that contained many genes and were highly significant (Figure 1A). Because we wanted to determine if there were abnormalities, we selected targets for investigation that were not differentially expressed in the RNA sequencing dataset, as this could impact our ability to observe morphological changes. Therefore, we first investigated the impact of hnRNP A1 loss on the nuclear envelope using Lamin B to assess nuclear envelope morphology. Cells treated with non-targeting siRNA (siNEG) demonstrated two predominant, normal phenotypes, including a ring phenotype of Lamin B surrounding the edges of the nucleus or diffuse Lamin B staining throughout the nucleus (Figure 2A). Treatment of cells with siA1 knocked down hnRNP A1 expression (Figure S2) and increased the prevalence of abnormal Lamin B-staining phenotypes by a factor greater than two (Figure 2B,C). Abnormal Lamin B-staining phenotypes were defined based on previous publications [9,20,25,28,33] and included punctate (Lamin B ‘spots’ within the nucleus), invagination (lines of Lamin B staining within the nucleus), and incomplete (Lamin B ring did not entirely surround the nucleus). When examining individual phenotypes, we found that the normal ring phenotype significantly decreased with siA1 treatment, while the diffuse phenotype was comparable for the siA1 and non-targeting control siRNA (siNEG)-treated groups (Figure 2D). The punctate phenotype was similar in number, regardless of treatment (Figure 2E), while the proportion of cells with an invagination or incomplete phenotype significantly increased with hnRNP A1 knockdown. This suggests that these latter two phenotypes largely contributed to the overall increase in abnormal Lamin B phenotypes in our hnRNP A1 knockdown model. Increased abnormal Lamin B phenotypes demonstrate that loss of hnRNP A1 function alters nuclear envelope morphology.

### 3.3. Qualitative Phenotypes of Lamin B Staining Can Be Identified and Quantified Using Three-Dimensional (3D) Images

**Figure 3:**
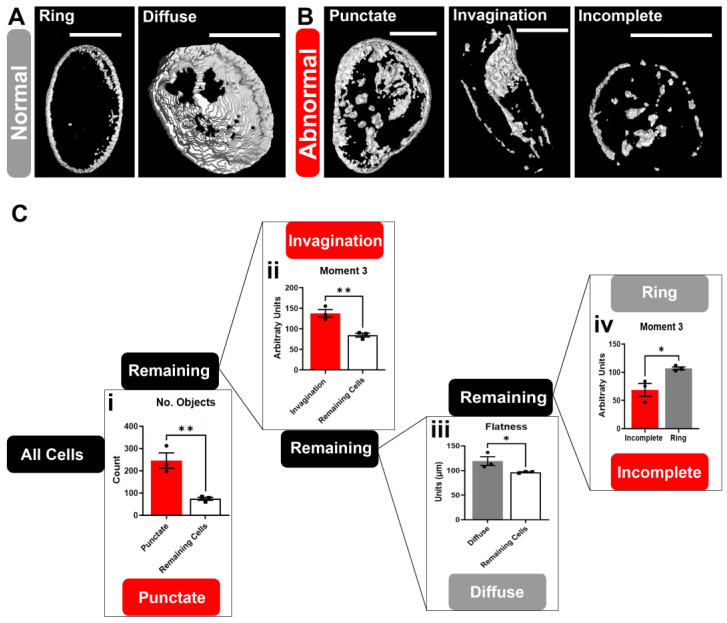
Establishing a method of automatic Lamin B phenotyping using 3D measurements. Three-dimensional renderings of Lamin B staining (white) were generated from a Z-stack of images. (**A**) The normal phenotypes, ring and diffuse, are visually distinct from the (**B**) abnormal phenotypes: punctate, invagination, and incomplete. Three-dimensional renderings were used to quantify each of the Lamin B phenotypes. (**C**) Schematic demonstrating how the five different phenotypes were separated and assigned to generate a novel automated phenotyping script. Cells were examined based on different 3D measurements (graphs) to separate normal (grey) and abnormal (red) phenotypes. After separating out an individual phenotype, the remaining cells (white) were assessed, based on another measurement, until all phenotypes could be mathematically grouped. Initially, the number of objects, which is the count of distinct, disconnected Lamin B objects in the cell, was used to define the punctate phenotype (**i**). Next, moment 3, a quantification of the 3D shape of Lamin B staining, was used to identify the invagination phenotype (**ii**). The remaining population was examined based on flatness, which is the depth of the staining in the third (z) dimension, and which defined the diffuse phenotype (**iii**). The last two phenotypes, ring and incomplete, could be separated based on their moment-3 measurement (**iv**). Scale bars = 10 µm, n = 3 replicates. Data are normalized to the values in the previous step (connecting node in **C**) within replicates and plotted as the mean ± SEM. Two-tailed independent *t*-tests were used to compare means between groups, as the expected effect was unknown, * *p* < 0.05, ** *p* < 0.01.

Next, we sought to define and measure Lamin B phenotypes quantitatively. As proof of principle, we developed a pipeline to build three-dimensional (3D) renderings from Z-stack images to quantify Lamin B-staining phenotypes. Three-dimensional images revealed that the ring and diffuse phenotypes were qualitatively similar, as both had a ring of staining around the nucleus. However, cells with a diffuse phenotype had more staining on the dome-shaped area surrounding the edge of the nucleus, whereas the ring phenotype had less staining on the dome (Figure 3A, Videos S1 and S2). The abnormal phenotypes in Figure 3B, Videos S3–S5 were qualitatively distinct from the normal phenotypes in Figure 3A. After finding observable differences in the 3D images between normal and abnormal phenotypes, we collected 3D measurements to generate an automatic method to assign Lamin B phenotypes within cells to reduce bias and generate similar results regardless of data quantification and experimental conditions, as well as decrease the time associated with manually identifying phenotypes.

Three-dimensional images of Lamin B staining enabled the collection of several measurements used to define the quantitative characteristics of the manually assigned phenotypes. A mathematical approach was developed using the known phenotypes (ring, diffuse, punctate, invagination, and incomplete) to automatically assign phenotypes based on subtractive selection across quantitative measurements using siNEG cells (Figure 3C). The automatic phenotyping process is illustrated in Figure 3C, where cells were initially grouped and examined based on the number of objects within the cell (i in Figure 3C). More objects within a cell corresponded to cells with punctate phenotypes. A threshold was established, wherein cells with an object count 3.3× the average of siNEG cells not currently assigned a phenotype were defined as having a punctate phenotype. This threshold was determined by taking the fold change in i of Figure 3C and making precise adjustments to determine the optimal value, 3.3, when the maximum number of punctate cells were correctly identified using the quantitative measurement. These cells, identified as punctate staining, were removed from the group based on the number of distinct objects stained for Lamin B. The remaining cells were then examined using moment 3, a 3D measurement of the 3D shape of the staining to identify cells with an invagination phenotype (ii in Figure 3C). The remaining cells were assessed based on their flatness, which examines the depth of the nucleus (iii in Figure 3C). This allowed for the separation of diffuse phenotype cells, as the diffuse phenotype has a hollow hemisphere of Lamin B staining, whereas the remaining cells had less depth of staining in the z-dimension. This left the ring and incomplete phenotypes as the remaining cells to be defined. To do this, moment 3 was again used to separate incomplete from ring, the latter of which had a higher moment-3 measurement than the incomplete phenotype (iv in Figure 3C).

To validate our automated system, we generated three new replicates of siNEG- and siA1-treated cells stained for Lamin B to analyze using our phenotyping program. The first replicate was used to compare the manual method of separating cells into normal- and abnormal-staining phenotypes and the automatic phenotyping program. Here, we found no differences in the number of cells classified as normal or abnormal between the two methods (Figure S3A,B). The same trend, no differences between the manual or computer-based methods, was found when examining the separate treatment groups, siNEG- (Figure S3A,B) and siA1- treated cells alone (Figure S3A,B). After confirming there were no differences between the manual and computer-based phenotyping methods, we used the latter to quantify the percent of cells with Lamin B abnormalities in siNEG- and siA1-treated cells (Figure S3C). We observed that significantly more cells in the siA1-treatment group demonstrated abnormal Lamin B staining compared to siNEG-treated cells, thus confirming our previous observations but with an unbiased methodology.

### 3.4. Nuclear Envelope Integrity Remains Intact with hnRNP A1 Knockdown

**Figure 4:**
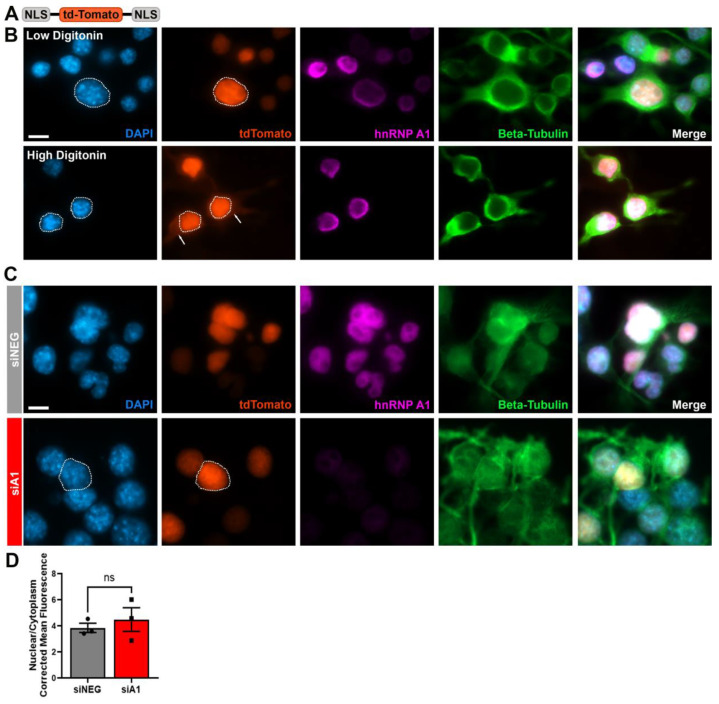
Nuclear envelope integrity is not compromised by hnRNP A1 knockdown. (**A**) Schematic of the 2xNLS-tdTomato plasmid used for the nuclear envelope integrity assay. (**B**) Cells were treated with a low concentration of digitonin (low digitonin; 20 µg for 4 min) or a high concentration of digitonin (high digitonin; 40 µg for 10 min) as controls, to visualize tdTomato localization patterns. In the low-digitonin condition, only plasma membrane is permeabilized and therefore, the tdTomato signal was predominantly nuclear. High concentrations of digitonin permeabilized the nuclear envelope and induced tdTomato leakage into the cytoplasm, indicated by tdTomato signal within the cytoplasm (arrows). (**C**) Comparison of tdTomato localization signal in siNEG- and siA1-treated cells. Dotted lines outline the nucleus, which were created using the corresponding DAPI image. There were no changes in cellular distribution of tdTomato signal in siNEG- compared to siA1-treated cells. (**D**) Quantification of the nuclear-to-cytoplasmic ratio of tdTomato signal showing no quantitative difference between siNEG- and siA1-treated cells. Data are graphed as mean ± SEM. Scale bars = 10 µm, n = 3 replicates. As siA1 was expected to produce a specific directional effect, a one-tailed independent *t*-test was used to compare means between siNEG and siA1 in (**D**), ns = not significant.

Because hnRNP A1 knockdown induced abnormal Lamin B phenotypes, and thus nuclear envelope morphology changes, we sought to assess the integrity of the nuclear envelope following hnRNP A1 knockdown. For this, we used a plasmid that contained two nuclear localization sequences (2xNLS-tdTomato) and performed co-transfection with siNEG or siA1 (Figure 4A). As experimental controls, digitonin (Dig) was used at a low concentration that would not compromise the nuclear envelope and a high concentration to permeabilize the nuclear envelope. In control cells treated with a low level of digitonin as a negative control, tdTomato signal was predominantly located within the nucleus, as expected (Figure 4B). Cells treated with a higher concentration of digitonin displayed cytoplasmic tdTomato, demonstrating that holes in the nuclear envelope induced leakage of tdTomato out of the nucleus (Figure 4B). In cells treated with siNEG or siA1, tdTomato signal appeared exclusively nuclear (Figure 4C). The ratio of tdTomato fluorescence in the nucleus to the cytoplasm was similar between the two treatment groups (Figure 4D), indicating that while hnRNP A1 knockdown negatively impacts nuclear envelope morphology, it does not compromise nuclear envelope integrity.

### 3.5. hnRNP A1 Knockdown Significantly Alters Components of NCT

**Figure 5:**
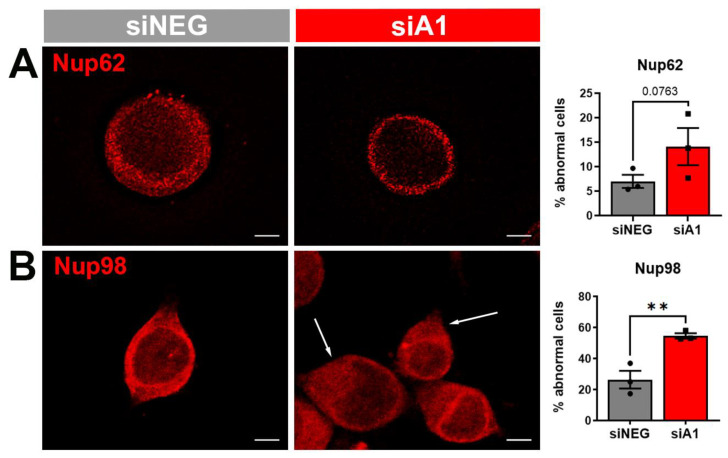

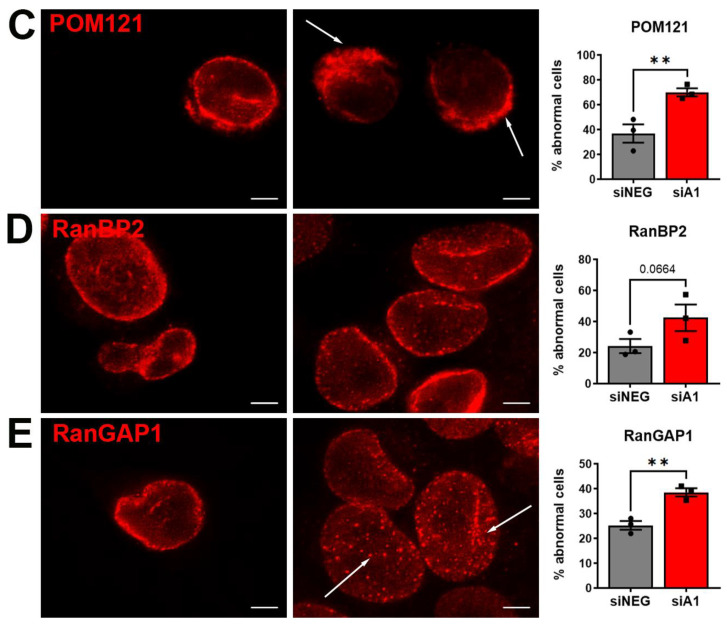
hnRNP A1 knockdown induces phenotypic changes in several components of NCT, including Nups. Cells treated with siNEG or siA1 were evaluated for phenotype abnormalities in several components of NCT, including (**A**) Nup62 and (**B**) Nup98, both part of the central channel of the NPC, (**C**) POM121, a component of the NPC transmembrane ring, (**D**) RanBP2 within the cytoplasmic ring and filaments, and (**E**) RanGAP1, which is involved in transport. Cells treated with siA1 showed an increase in the number of cells with Nup98, POM121, and RanGAP1 phenotype abnormalities as compared to siNEG. Arrows point to different features defined as abnormal for each marker. No significant differences in the number of cells with alterations were found for Nup62 or RanBP2 between the groups. Representative images for each marker were generated by deconvolving 40X Z-stack images. Data are graphed as the mean ± SEM. Scale bars = 5 µm, n = 3 replicates. As siA1 was expected to produce a specific directional effect, a one-tailed independent *t*-test was used to compare means between siNEG and siA1, ** *p* < 0.01.

Next, we evaluated several markers of the NPC and transport, as GO analyses identified enrichment for these terms following hnRNP A1 knockdown. First, we examined markers for different compartments of the NPC which have been shown to been altered in neurologic diseases, including Nup62 and Nup98 in the central channel, POM121 to demarcate the transmembrane ring, and RanBP2 within the cytoplasmic ring and filaments. Impaired Nups, including changes in localization patterns, can disrupt the structure and function of the NPC [9,24]. Based on previous findings about distribution patterns, we evaluated cells treated with siNEG and siA1 to identify alterations in these markers [17]. Nup62 demonstrated a robust phenotype that surrounded the nucleus, which is categorically normal, in both siNEG- and siA1-treated cells, and there was no significant difference in the number of abnormal cells between the two groups (Figure 5A). Nup98, another component of the central channel, was predominantly ring-shaped around and within the nucleus in the control condition, which was quantified as a normal phenotype (Figure 5B). Other neurodegenerative disease models have documented abnormalities in Nup98 such as reduced expression, cytoplasmic mislocalization, and possible nuclear membrane invaginations, all of which were observed at a higher proportion in cells treated with siA1 (Figure 5B). Further, we found a significant decrease in Nup98 protein expression levels following hnRNP A1 knockdown (Figure S3), aligning with the previous literature that shows reduced expression levels of Nup98 in disease models [34]. POM121 demonstrated a similar normal phenotype as Nup98, with staining around and within the nucleus in control cells (Figure 5C). Abnormal POM121 phenotypes, including cytoplasmic mislocalization with or without a ring around the nucleus and invaginations [17], were observed in both siNEG and siA1 groups, but were significantly increased in the siA1-treated cells (Figure 5C). Although we found significant alterations in Nup98 and POM121 following hnRNP A1 knockdown, we observed no significant changes in RanBP2, a component of the NPC cytoplasmic ring and filaments (Figure 5D). While we noted the presence of normal RanBP2 ring phenotypes as well as abnormal cytoplasmic staining, the number of cells with an abnormal phenotype was not significantly different between siNEG- and siA1-treated cells (Figure 5D). Finally, we examined RanGAP1, a nucleocytoplasmic transport protein which is normally distributed around the nucleus and, to a lesser extent, within the cytoplasm [35,36]. Here, we noted that cells treated with siA1 showed abnormal distribution patterns of RanGAP1, including increased amounts within the nucleus, and which were largely a punctate phenotype (Figure 5E). Quantification revealed a significant increase in the number of cells with RanGAP1 abnormalities in siA1 cells compared to siNEG.

### 3.6. hnRNP A1 Knockdown Alters Active NCT

**Figure 6:**
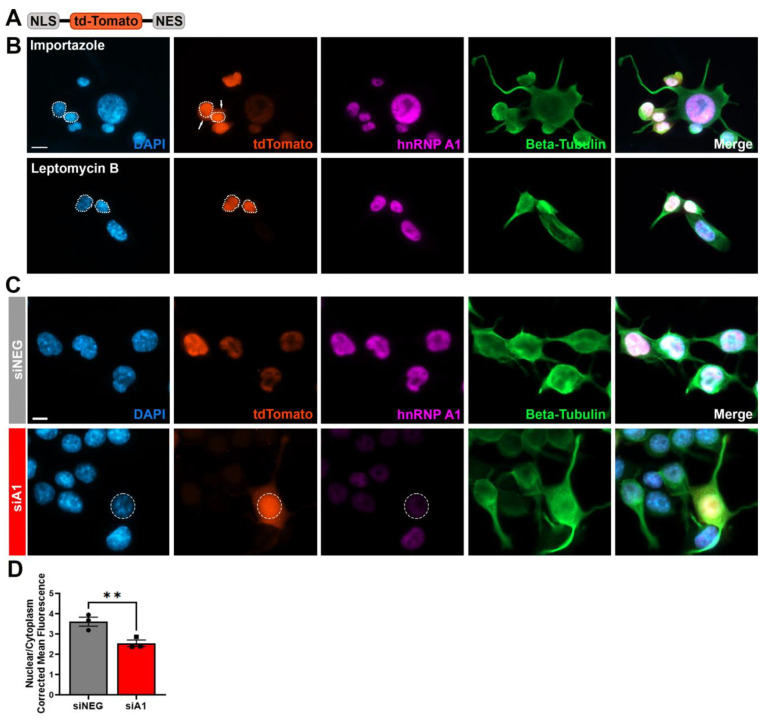
hnRNP A1 knockdown leads to deficits in active NCT. (**A**) Schematic of the S-tdTomato plasmid used to evaluate active NCT. (**B**) Cells were treated with Importazole, a nuclear import inhibitor or Leptomycin B, a nuclear export inhibitor to visualize localization of tdTomato signal. As a nuclear import inhibitor, Importazole induced cytoplasmic accumulation of tdTomato signal (arrows) while Leptomycin, a nuclear export inhibitor, led to nuclear localization of tdTomato. Dotted lines outline the nucleus, which were created using the corresponding DAPI image. Scale bar = 20 µm. (**C**) Representative images of cells co-transfected with S-tdTomato and siNEG or siA1. siNEG cells showed nuclear localization of tdTomato signal, while cells treated with siA1 demonstrated cytoplasmic localization of tdTomato signal. Dotted lines outline the nucleus, which were created using the corresponding DAPI image. (**D**) Quantification of the nuclear-to-cytoplasmic ratio of tdTomato signal, showing a significant decrease in the nuclear/cytoplasmic ratio in cells treated with siA1. Scale bar = 10 µm, n = 3 replicates. Data are plotted as mean ± SEM. As siA1 was expected to produce a specific directional effect, a one-tailed independent *t*-test was used to compare means between siNEG and siA1 in (**D**), ** *p* < 0.01.

While we showed that loss of hnRNP A1 function leads to alterations in NCT components, such as Nups and RanGAP1, these are not indicative of functional NCT changes. Therefore, we examined functional changes associated with changes in components of NCT by investigating active NCT. To do this, we performed co-transfection of siRNA with a plasmid (Shuttle-tdTomato [S-tdTomato]) containing a nuclear localization signal (NLS), a nuclear export signal (NES), and a fluorescent marker (Figure 6A), a plasmid commonly used to assess NCT alterations [25,34,37,38]. S-tdTomato is continuously shuttled between the nucleus and cytoplasm through active transport. The NLS is more potent than the NES, so higher levels of tdTomato fluorescence were expected to be found in the nucleus under control conditions. To validate the transport patterns of S-tdTomato, cells were treated with a nuclear import inhibitor (importazole) or nuclear export inhibitor (leptomycin B) (Figure 6B). Importazole, as a nuclear import inhibitor, prevented import of S-tdTomato and induced cytoplasmic accumulation of tdTomato signal (Figure 6B). Conversely, leptomycin B, a nuclear export inhibitor prevented export of S-tdTomato leading to nuclear accumulation of tdTomato signal (Figure 6B). After establishing that this assay is capable of identifying changes in NCT, we examined cells treated with siNEG or siA1 (Figure 6C). Cells treated with siNEG demonstrated predominantly nuclear tdTomato localization quantitated by a high nuclear-to-cytoplasmic fluorescence ratio (Figure 6D). Conversely, treatment with siA1 caused a significant increase in tdTomato cytoplasmic signal, indicated by a decrease in the nuclear-to-cytoplasmic ratio of the fluorescence signal (Figure 6D). This indicates that structural alterations in NCT due to hnRNP A1 knockdown also have a functional consequence, in that active NCT across the nuclear envelope is compromised.

### 3.7. Lamin B Is Significantly Altered in Neurons from MS as Compared to Healthy Controls

**Figure 7:**
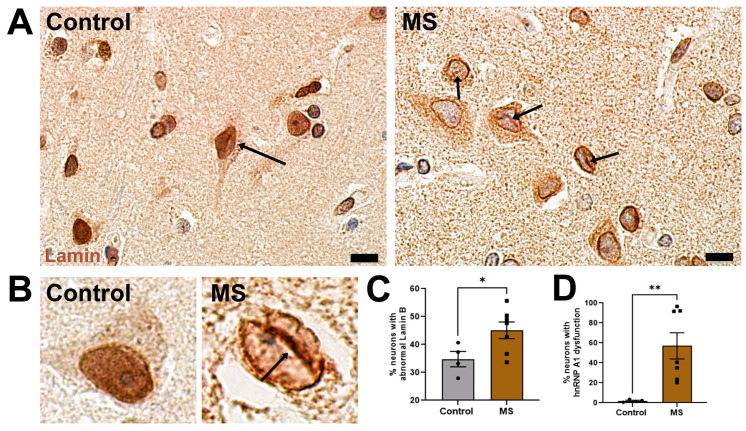
Lamin B phenotype abnormalities are prevalent in MS tissues. (**A**) Control and MS grey matter stained for Lamin B (brown) and hematoxylin (blue). Arrows point to normal (control) and abnormal (MS) phenotypes. (**B**) Higher magnification of neurons from control and MS grey matter illustrating phenotypes from the respective groups. Neurons from MS samples demonstrate Lamin B invagination phenotypes (arrow) among other abnormal phenotypes. (**C**) Quantification of the percent of neurons exhibiting abnormal Lamin B phenotypes and (**D**) hnRNP A1 dysfunction in cortex from control and MS samples. Data are plotted as mean ± SEM. Scale bars = 10 µm, n = 4 control and n = 7 MS cases. The percent of abnormal phenotypes in human samples was normally distributed (Shapiro–Wilk W: Control W = 0.9232, *p* = 0.5505; MS W = 0.9811, *p* = 0.9877) and the variance between the two samples was statistically equal (F-test: F_11,4_ = 1.863, *p* = 0.5761). Therefore, an independent *t*-test was used to analyze the samples, * *p* < 0.05.

Lastly, we wanted to validate our findings in the MS brain, as previous publications have demonstrated neuronal hnRNP A1 dysfunction [5,6,21]. Many neurodegenerative diseases with RBP dysfunction have documented morphological changes in Lamin B indicative of broader alterations in NCT [9,17,20,23]. Human brain tissue samples were age, sex, and brain-region matched (Table S1). In control tissue, Lamin B predominantly formed a ring of staining around neuronal nuclei with diffuse staining within the nucleus (Figure 7A,B), which we also observed as normal phenotypes in our in vitro system. In controls, a smaller portion of neurons demonstrated abnormal Lamin B phenotypes, including an invagination phenotype (Figure 7A,B), similar to what was observed in our hnRNP A1 knockdown model, as well as in the literature [17,20,23]. In MS cases, however, the occurrence of abnormal Lamin B-staining phenotypes was significantly increased as compared to controls (Figure 7C) in MS cases with neuronal hnRNP A1 dysfunction (Figure 7D), thus validating a portion of our findings from the in vitro model in human disease.

## 4. Discussion

Dysfunctional RBPs and NCT machinery are now considered hallmarks of neurodegenerative disease [16,39]. Interestingly, it has been hypothesized that one may cause the other and vice versa. For example, damage to the NCT machinery can alter homeostatic NCT and induce or exacerbate the improper functioning of RBPs and their cytoplasmic mislocalization. Alternatively, dysfunctional RBPs can lead to changes in the NCT machinery [9]. Both scenarios introduce a loop, wherein altered RBPs and NCT machinery contribute to the dysfunction of one another and exacerbate the detrimental effects of each. Thus, changes in one or both may represent a possible mechanism of neurodegeneration in neurodegenerative diseases.

Here, we show that dysfunction of the RBP, hnRNP A1, modelled through its knockdown, which mimics a loss of homeostatic function in neurodegenerative diseases, results in alterations to the NCT machinery, including perturbations in nuclear envelope morphology, abnormalities in Nups and RanGAP1, a nuclear transport receptor, and changes in active NCT. First, using differentially expressed genes from a previously published RNA sequencing dataset employing siNEG- and siA1-treated Neuro-2A cells, we identified enrichment of NCT machinery terms, including nuclear envelope and lamina, NPC, and transport. Given the published relationship between dysfunctional RBPs and NCT and these findings, we further investigated the impact of hnRNP A1 dysfunction on different aspects of NCT machinery.

Nuclear envelope and lamina were two highly enriched GO terms prompting us to investigate Lamin B changes following hnRNP A1 knockdown. In control cells, we found that the majority of cells exhibited normal Lamin B phenotypes. However, in siA1-treated cells, there was an increased prevalence of abnormal phenotypes, including incomplete and invaginated Lamin B patterns. The noted normal and abnormal Lamin B phenotypes are well-documented in the literature [9,17,20,23,24]. Thus, our defined phenotypes are in line with other publications that denote Lamin B abnormalities and phenotypic changes in models of neurodegenerative disease, including those with significant RBP dysfunction. First, we manually binned cells into normal- and abnormal-phenotype groups and determined that hnRNP A1 knockdown significantly increased the number of cells with Lamin B abnormalities. We then developed an automated computer script to perform this function to create a uniform, unbiased process of assigning phenotypes to cells. The computer script confirmed our initial findings, and we found no significant differences in quantification between manual and automatic phenotyping methods. Interestingly, abnormal nuclear morphology including nuclear envelope invaginations, occurs in laminopathies and can be indicative of changes in the lamin nucleoskeleton [40]. Lamin B mutants induce changes in localization patterns, DNA damage, and eventual apoptosis in neurons, indicating that morphological changes may be functionally important [23]. Further, lamins, specifically Lamin B, are important for NPC distribution within the nuclear envelope [18]. Thus, it is possible that Lamin B abnormalities may also impact the distribution and number of NPCs within the envelope.

Next, we investigated several other NCT machinery proteins that were not differentially expressed in the RNA sequencing dataset, to determine the effect of hnRNP A1 knockdown on morphology. We selected markers from different substructures within the NPC to achieve an overview of the NPC as well as RanGAP1, which functions as a nuclear transport receptor. Here, we found significant alterations in Nup98, POM121, and RanGAP1, but not Nup62 or RanBP2. The identified normal and abnormal phenotypes were defined based on their prevalence in the siNEG group, as well as previously documented changes [17]. We noted that abnormalities in Nup98 and POM121, such as cytoplasmic mislocalization and altered distribution at the nuclear envelope, were increased with hnRNP A1 knockdown. Interestingly, Nup98 is essential for proper NPC assembly, in that knockout of Nup98 affects the ability of certain Nups to form functional NPCs [41]. This creates the possibility that abnormal distribution of Nups like Nup98 may also impact NPC function. Additionally, we found increased RanGAP1 within the nucleus following siA1 treatment. RanGAP1 normally surrounds the nuclear envelope and is within the cytoplasm, and assists with transport of proteins from the cytoplasm to the nucleus. Accumulation and aggregation of nuclear RanGAP1 is abnormal and suggests that it is no longer capable of efficiently transporting cargo into the nucleus. Disrupted distribution of RanGAP1 has been previously observed in neurodegenerative disease, and may be indicative of defects in NCT [36,37]. Our findings further indicate that not all components of the NCT machinery are changed by hnRNP A1 knockdown, which aligns with other studies where different models present changes in some, but not all, NCT components [9,34,38]. This could be due to the type of RBP dysfunction model being employed, such as knockdown compared to aggregation or mislocalization models. Future studies may seek to investigate the impact of hnRNP A1-derived mutations on the NCT machinery to determine if there are commonalities.

We next investigated functional changes in active NCT using the NLS-tdTomato-NES plasmid, a commonly used reporter, to examine the impact on transport by evaluating the nuclear-to-cytoplasmic ratio of tdTomato signal [25,34,37,38]. We found that hnRNP A1 knockdown significantly altered NCT, wherein there was an increase in tdTomato cytoplasmic signal with siA1 treatment compared to siNEG treatment. NCT is critical for cellular homeostasis, and small changes in this process may impact the ability of macromolecules to properly traffic to locations within the cell, leading to detrimental consequences. Further, a commonality amongst neurodegenerative diseases is disrupted NCT, which may suggest that underlying RBP dysfunction in these diseases initiates or exacerbates problems with transport.

After determining that hnRNP A1 knockdown impacted multiple aspects of NCT, we sought to validate our findings in human tissues where hnRNP A1 dysfunction is observed. Using Lamin B to demarcate overall nuclear envelope and lamina morphology, we found a significant increase in abnormalities in neurons from MS tissues, which exhibit hnRNP A1 dysfunction [6], as compared to controls. This demonstrates a clinical relevance of findings related to nuclear structure and suggest that, similarly to other neurodegenerative diseases, NCT machinery perturbations may contribute to neuronal degeneration in MS and may be closely related to RBP dysfunction.

It is well-documented that NPC alterations, including loss, aggregation, and dysfunction of Nups, nuclear transport receptor abnormalities, and nuclear envelope or morphology changes can impact overall NCT and, therefore, cell function [9,36,42,43]. Therefore, altered NCT represents a possible mechanism of neuronal damage caused by hnRNP A1 dysfunction. It has been previously demonstrated that hnRNP A1 knockdown decreases neuronal viability, measured through changes in neurite length, and is associated with increased cytotoxicity [22]. Altered NCT interferes with homeostatic RNA nuclear export and translation, which has been linked to neurodegenerative disease [14]. RNA accumulation in the nucleus, in addition to hnRNP A1 dysfunction, could cause differential RNA expression in MS and models of hnRNP A1 dysfunction [21,22]. Differential gene expression induced by hnRNP A1 dysfunction and altered NCT caused by hnRNP A1 knockdown may lead to increased cellular damage. Additionally, changes in NCT due to hnRNP A1 knockdown could impact other RBPs that undergo nuclear import, such as TDP-43, leading to their dysfunction, as is observed in ALS and FTLD. Modulating import and export proteins can prevent toxicity associated with dysfunctional RBPs [25,44,45]. Therefore, altering the efficacy of NCT can prevent RBP mislocalization and rescue toxic effects associated with its mislocalization and deficient nuclear import. Future research will be needed to detect the mechanistic contribution of hnRNP A1 dysfunction and altered NCT to neuronal health and viability.

In summary, we demonstrated that dysfunctional hnRNP A1, modelled through its knockdown, caused significant perturbations in nuclear envelope morphology, NCT machinery proteins, including Nups and transport receptors, and NCT. In addition, we developed a novel mathematical method using 3D images of Lamin B staining to quantify and automatically identify normal and abnormal phenotypes of Lamin B staining. This method will reduce bias and provide a method to assess these changes uniformly. HnRNP A1 dysfunction changes in the NCT machinery represent a potential mechanism of neuronal damage and provide a novel avenue of investigation to understand neurodegenerative mechanisms in MS.

## Data Availability

The computer script for the novel phenotyping method is available through open access on GitHub.

## Supplementary Materials

The following supporting information can be downloaded at: https://www.mdpi.com/article/10.3390/brainsci14101039/s1. Figure S1: siA1 induces concomitant protein changes in differentially expressed RNA sequencing targets. Several RNA targets identified to be differentially expressed via RNAseq following hnRNP A1 knockdown were examined for concomitant changes in protein expression. Cells treated with siA1 showed significant hnRNP A1 knockdown compared to siNEG treated cells. From these same samples, hnRNP A1 knockdown also significantly impacted RanGAP1 and transportin-1 protein expression levels. Data are plotted as mean ± SEM with n = 3 replicates, one-tailed independent *t*-test, * *p* < 0.05 ** *p* < 0.01 **** *p* < 0.0001. Figure S2: siA1 significantly decreases hnRNP A1 expression compared to siNEG in Neu-ro-2a cells. Cells treated with siA1 showed significant hnRNP A1 (green) knockdown compared to siNEG treated cells. Data are plotted as mean of one replicate of the experiment. Scale bar = 20 µm, n = 3 replicates, one-tailed, paired, ratio *t*-test, * *p* < 0.05. Figure S3: Contingency tables and graphs revealing no difference between the automatic and manual Lamin B phenotyping methods. (A) The number of cells with normal or abnormal Lamin B phenotypes were compared between the manual and automatic quantification methods in all cells (cells from both siNEG and siA1 groups), siNEG cells alone, and siA1 cells alone. (B) Statistical tests demonstrate a lack of differences between the two methods all cells (black), siNEG cells alone (grey), and siA1 cells lone (red). A power analysis found that a sample size of 5817 is needed to achieve a power of 0.8 due to the small effect size. With a sample size that large any detected differences would be biologically irrelevant. The small effect size also indicates the certainty that the two groups are not different. (C) Quantification of Lamin B phenotypes using the automatic computer script demonstrating a significant increase in the percent of cells with abnormal staining in the siA1 group. Data in (A) are plotted as the number of cells exhibiting a normal or abnormal phenotype with n = 1 replicate for both manual and automatic methods. Data in (C) are plotted as mean ± SEM with n = 3 replicates, one-tailed independent *t*-test, ** *p* < 0.01. Figure S4: Nup98 protein expression is significantly decreased following hnRNP A1 knockdown. Western blots for hnRNP A1, Nup98, and Beta-actin in siNEG and siA1 treated cells. Three individual replicates are shown for each condition. One representative actin blot is shown. Quantification of hnRNP A1 band density levels confirmed knockdown with siA1 treatment and changes in Nup98 expression levels following hnRNP A1 knockdown. Relative band density levels were normalized to beta-actin. Data is plotted as the mean ± SEM, n = 3 replicates, paired *t*-test ** *p* < 0.01. Table S1: Characteristics of the human samples used in the study. Sex, age, diagnosis, cortical area from which tissue was obtained, and post-mortem delay for each individual human sample used in this study. M = male, F = female, MS = Multiple sclerosis, PP = primary progressive, SP = secondary progressive. Video S1: Diffuse phenotype of Lamin B staining in 3D. The staining is a dome around the nucleus. The center is hollow and devoid of Lamin B staining forming a hollow semi-circle object. Scale bar = 10 µm. Video S2: Ring phenotype of Lamin B staining in 3D. The staining forms a ring around the nucleus, with a small ‘cap’ of staining at the top of the nuclear envelope. Scale bar = 10 µm. Video S3: Invagination phenotype of Lamin B staining in 3D. The outer ring is not smooth and has an infolding near the top of the nucleus. Invaginations of Lamin B may or may not be connected to the outer ring. Scale bar = 10 µm. Video S4: Punctate phenotype of Lamin B staining in 3D. A dome of staining is present like the diffuse phenotype. However, the center of the nucleus is filled with specks of Lamin B staining. Scale bar = 10 µm. Video S5: Incomplete phenotype of Lamin B staining in 3D. A ring of staining is present around the majority of the nucleus. However, a lack of staining is apparent in one section around the nucleus. Scale bar = 10 µm.
