# Supplementary material for: Heterogeneous Nuclear Ribonucleoprotein A1 Knockdown Alters Constituents of Nucleocytoplasmic Transport
Source: Brain Sci. 2024 Oct 19;14(10):1039. doi: 10.3390/brainsci14101039 (PMC11505608; doi:10.3390/brainsci14101039)
Supplement: Supplementary file 1 [file brainsci-14-01039-s001.zip › Supplementary Material Legends.pdf]

Figure S1: siA1 induces concomitant protein changes in differentially expressed RNA sequencing targets. Several RNA targets identified to be differentially expressed via RNAseq following hnRNP A1 knockdown were examined for concomitant changes in protein expression. Cells treated with siA1 showed significant hnRNP A1 knockdown compared to siNEG treated cells. From these same samples, hnRNP A1 knockdown also significantly impacted RanGAP1 and transportin-1 protein expression levels. Data are plotted as mean ± SEM with n=3 replicates, one-tailed independent t-test, *p<0.05 **p<0.01 ****p<0.0001.

Figure S2: siA1 significantly decreases hnRNP A1 expression compared to siNEG in Neu-ro-2a cells. Cells treated with siA1 showed significant hnRNP A1 (green) knockdown compared to siNEG treated cells. Data are plotted as mean of one replicate of the experiment. Scale bar=20 µm, n=3 replicates, one-tailed, paired, ratio t-test, *p<0.05.

Figure S3: Contingency tables and graphs revealing no difference between the automatic and manual Lamin B phenotyping methods. (A) The number of cells with normal or abnormal Lamin B phenotypes were compared between the manual and automatic quantification methods in all cells (cells from both siNEG and siA1 groups), siNEG cells alone, and siA1 cells alone. (B) Statistical tests demonstrate a lack of differences between the two methods all cells (black), siNEG cells alone (grey), and siA1 cells lone (red). A power analysis found that a sample size of 5817 is needed to achieve a power of 0.8 due to the small effect size. With a sample size that large any detected differences would be biologically irrelevant. The small effect size also indicates the certainty that the two groups are not different. (C) Quantification of Lamin B phenotypes using the automatic computer script demonstrating a significant increase in the percent of cells with abnormal staining in the siA1 group. Data in (A) are plotted as the number of cells exhibiting a normal or abnormal phenotype with n=1 replicate for both manual and automatic methods. Data in (C) are plotted as mean ± SEM with n=3 replicates, one-tailed independent t-test, **p<0.01.

Figure S4: Nup98 protein expression is significantly decreased following hnRNP A1 knockdown. Western blots for hnRNP A1, Nup98, and Beta-actin in siNEG and siA1 treated cells. Three individual replicates are shown for each condition. One representative actin blot is shown. Quantification of hnRNP A1 band density levels confirmed knockdown with siA1 treatment and changes in Nup98 expression levels following hnRNP A1 knockdown. Relative band density levels were normalized to beta-actin. Data is plotted as the mean ± SEM, n=3 replicates, paired t-test *p<0.05. **p<0.01.

Video S1: Diffuse phenotype of Lamin B staining in 3D. The staining is a dome around the nucleus. The center is hollow and devoid of Lamin B staining forming a hollow semi-circle object. Scale bar=10µm.

Video S2: Ring phenotype of Lamin B staining in 3D. The staining forms a ring around the nucleus, with a small ‘cap’ of staining at the top of the nuclear envelope. Scale bar=10µm.

Video S3: Invagination phenotype of Lamin B staining in 3D. The outer ring is not smooth and has an infolding near the top of the nucleus. Invaginations of Lamin B may or may not be connected to the outer ring. Scale bar=10μm.

Video S4: Punctate phenotype of Lamin B staining in 3D. A dome of staining is present like the diffuse phenotype. However, the center of the nucleus is filled with specks of Lamin B staining. Scale bar=10μm.

Video S5: Incomplete phenotype of Lamin B staining in 3D. A ring of staining is present around the majority of the nucleus. However, a lack of staining is apparent in one section around the nucleus. Scale bar=10μm.
