# Supplementary material for: Heterogeneous Nuclear Ribonucleoprotein A1 Knockdown Alters Constituents of Nucleocytoplasmic Transport
Source: Brain Sci. 2024 Oct 19;14(10):1039. doi: 10.3390/brainsci14101039 (PMC11505608; doi:10.3390/brainsci14101039)
Supplement: Supplementary file 1 [file brainsci-14-01039-s001.zip › Table S1.pdf]

Sample ID	Sex	Age	Diagnosis	Cortical Area	Post-mortem delay
Control 1	M	48	Control	Frontal	5.5
Control 2	M	57	Control	Frontal	4.42
Control 3	M	54	Control	Frontal	9.17
Control 4	F	54	Control	Parietal	8.00
MS 1	M	61	MS (PP)	Parietal	3.00
MS 2	M	46	MS (SP)	Frontal	8.17
MS 3	F	59	MS (SP)	Parietal	7.58
MS 4	F	70	MS (SP)	Frontal	6.92
MS 5	M	50	MS (SP)	Frontal	5.67
MS 6	F	47	MS (SP)	Parietal	5.33
MS 7	M	48	MS (SP/PP)	Parietal	7.83

Table S1 Characteristics of the human samples used in the study. Sex, age, diagnosis, cortical area from which tissue was obtained, and post-mortem delay for each individual human sample used in this study. M=male, F=female, MS=Multiple sclerosis, PP=primary progressive, SP=secondary progressive
