# Supplementary figures and images for: Heterogeneous Nuclear Ribonucleoprotein A1 Knockdown Alters Constituents of Nucleocytoplasmic Transport
Source: Brain Sci. 2024 Oct 19;14(10):1039. doi: 10.3390/brainsci14101039 (PMC11505608; doi:10.3390/brainsci14101039)

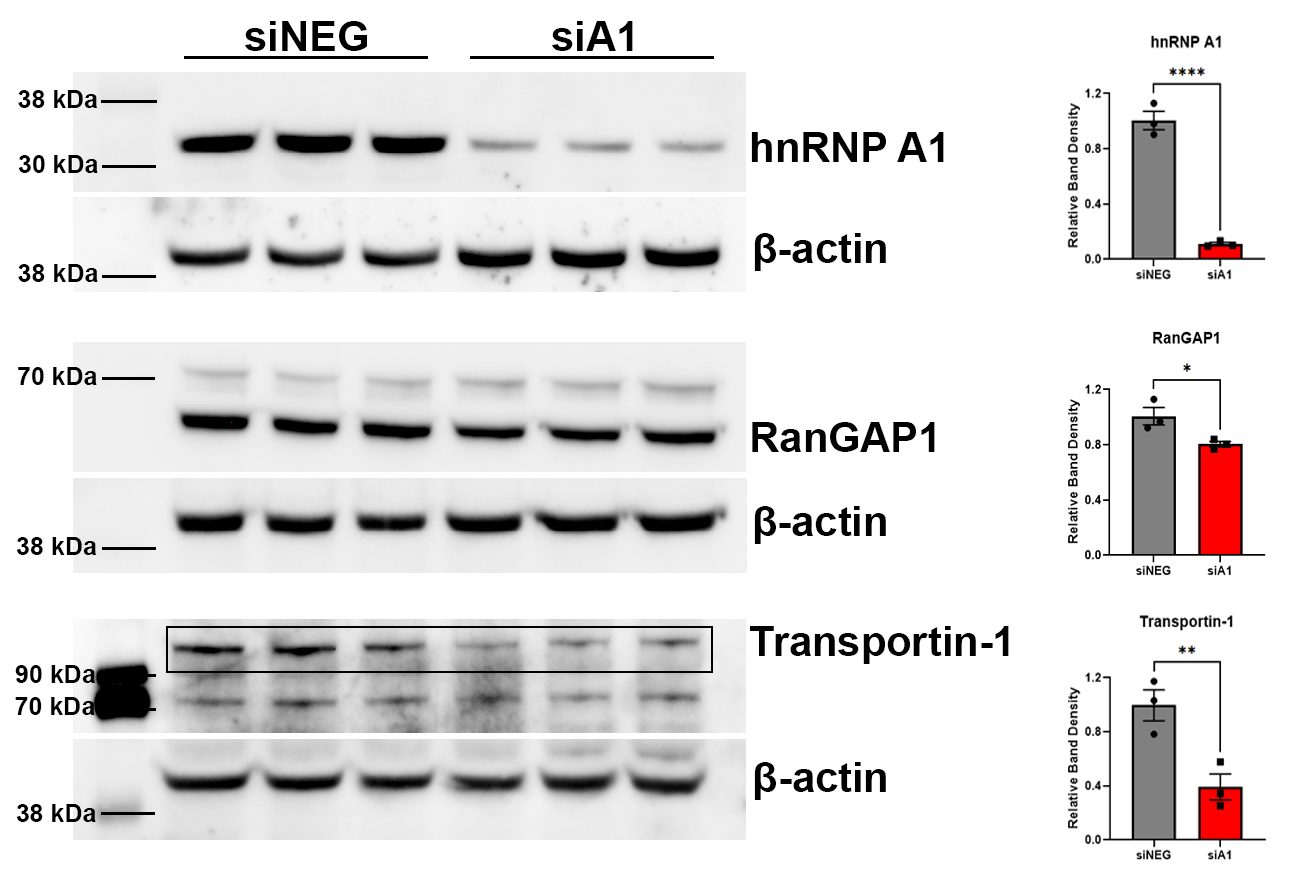

Supplement: Supplementary file 1 [file brainsci-14-01039-s001.zip › Figure S1.tif]

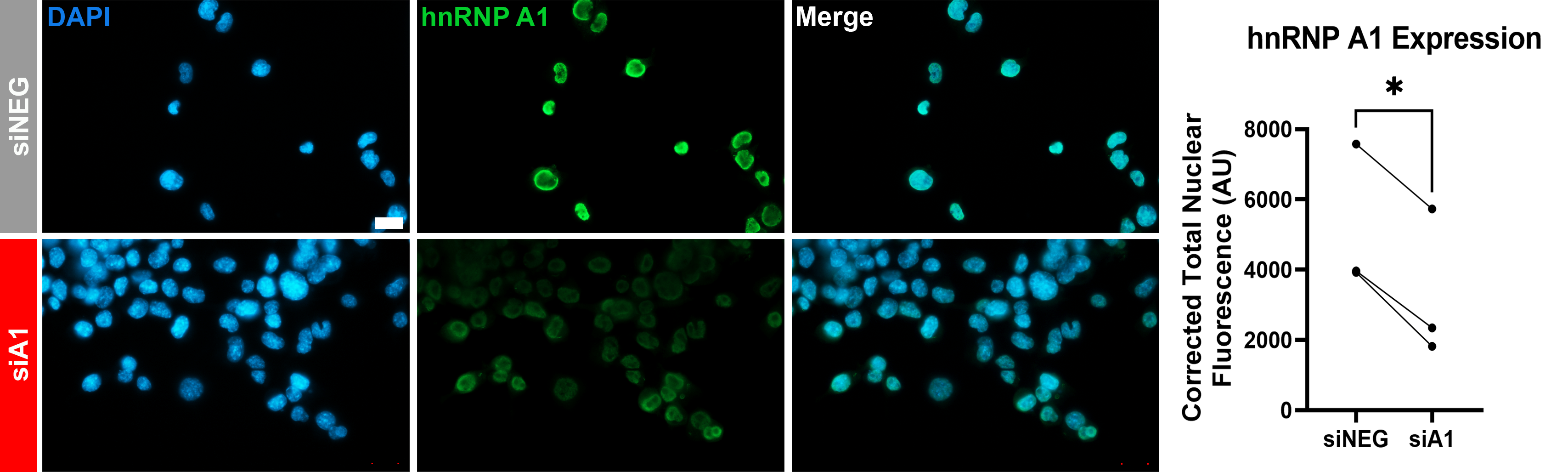

Supplement: Supplementary file 1 [file brainsci-14-01039-s001.zip › Figure S2.tif]

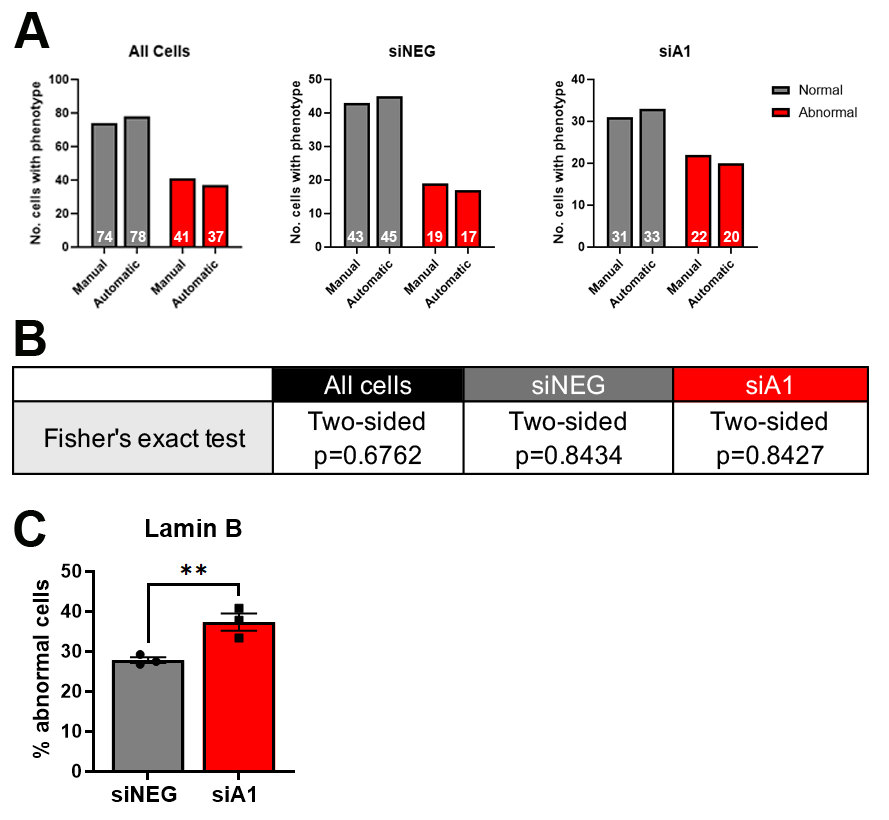

Supplement: Supplementary file 1 [file brainsci-14-01039-s001.zip › Figure S3.tif]

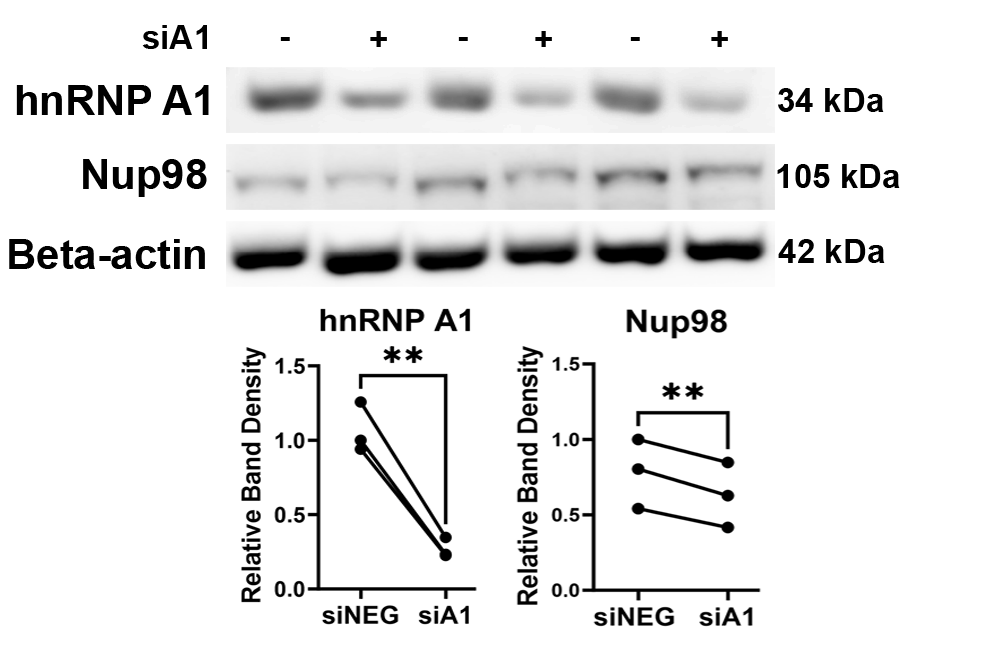

Supplement: Supplementary file 1 [file brainsci-14-01039-s001.zip › Figure S4.tif]
